# The right way to kiss: directionality bias in head-turning during kissing

**DOI:** 10.1038/s41598-017-04942-9

**Published:** 2017-07-14

**Authors:** A. K. M. Rezaul Karim, Michael J. Proulx, Alexandra A. de Sousa, Chhanda Karmaker, Arifa Rahman, Fahria Karim, Naima Nigar

**Affiliations:** 1 Department of Psychology, University of Dhaka, Dhaka, 1000 Bangladesh; 2 Envision Research Institute, 610 N. Main St, Wichita, KS 67203 USA; 3 The Smith-Kettlewell Eye Research Institute, 2318 Fillmore St, San Francisco, CA 94115 USA; 4 Department of Psychology, University of Bath, Bath, BA2 7AY UK; 5 Culture and Environment: Psychology, Bath Spa University, Bath, BA2 9BN UK

## Abstract

Humans have a bias for turning to the right in a number of settings. Here we document a bias in head-turning to the right in adult humans, as tested in the act of kissing. We investigated head-turning bias in both kiss initiators and kiss recipients for lip kissing, and took into consideration differences due to sex and handedness, in 48 Bangladeshi heterosexual married couples. We report a significant male bias in the initiation of kissing and a significant bias in head-turning to the right in both kiss initiators and kiss recipients, with a tendency among kiss recipients to match their partners’ head-turning direction. These interesting outcomes are explained by the influences of societal learning or cultural norms and the potential neurophysiological underpinnings which together offer novel insights about the mechanisms underlying behavioral laterality in humans.

## Introduction

It is well documented that humans have a bias for turning to the right in a number of settings. For example, most newborn infants prefer to lie with their heads turned to the right, rather than to the left^1^. Strikingly, even human fetuses prefer to turn their heads to the right during the final weeks of gestation at an age for freely moving the head^2, 3^. This preference is also exhibited by newborns of both vaginal and caesarean deliveries^4–6^ and maintained for the first six months of birth^2, 3^. The rightward bias in head-turning may occur in response to both aversive and nonaversive stimulation^7^ and is more frequent among the children of two right-handed parents^8^. This is one of the earliest behavioral asymmetries in humans and is thought to predict handedness^1, 2, 9–13^, and also affect the subsequent development of spatial-perceptual and motor preferences by increasing visual orientation to the right side^14–17^.

Empirical evidence of this effect has been demonstrated in a number of studies in both real life and artificial situations. For example, Güntürkün^18^ was the first to test this effect in 124 couples kissing (unless otherwise specified, kissing refers to lip-kissing in this paper) in public places like international airports, railway stations, beaches and parks in the United States, Germany and Turkey. He recorded the head-turning behavior of each couple during a single kiss and reported that 64.5% (80) of the couples turned their heads to the right and 35.5% (44) turned their heads to the left during kissing. Barrett, Greenwood and McCullagh^19^ studied laterality in both publicly kissing couples and in individual participants kissing dolls in a laboratory setting. In line with Güntürkün^18^, these researchers found that 80% of kissing couples and 77% of individual participants kissing dolls turned their heads to the right. More recent studies in a laboratory setting also consistently reported a rightward head-turning bias in kissing^20, 21^. A very recent study has suggested that laterality in kissing is contextual, showing a rightward head-turning bias in romantic couples (including parent-parent) and a leftward bias in a parent-child kissing situation^22^.

Theories of laterality have put forward a few postulates to explain the rightward head-turning bias in kissing. One of the postulates relates to the innate motor bias in head-turning^18, 19^. It posits that laterality in kissing in adult humans reflects the persistence of a bias for turning the head to the right that is present in fetuses and newborns^18^. A second postulate proposes that the head-turning bias in kissing is related to other sorts of lateralities, such as handedness and footedness^20, 21^. A more recent study has challenged both these postulates by investigating how cultural factors, such as a predominant reading or writing direction, contribute to laterality in kissing^23^. Using a cross-cultural approach that study examined head-turning direction in both kissing couples and doll kissing in a laboratory setting. It revealed that a significantly rightward head-turning bias during kissing was apparent in left-to-right readers (e.g., English) and a significantly leftward head-turning bias in right-to-left readers (e.g., Hebrew, Arabic)^23^. Thus the rightward bias in kissing that is observed in Western societies is turned into a leftward bias in Middle-Eastern societies. Based on this finding, Shaki^23^ suggested that the directional bias in head-turning can be shaped by cultural spatial habits, rather than reflecting an innate lateral asymmetry. Thus it appears that laterality in kissing is a complex issue and this complexity might result from the influence of socio-cultural pressures on behavioral laterality through interactions with its genetic endowment. Socio-cultural pressures can implicitly or explicitly force individuals to align their behavior to that of their peers within the population. In support of this, a recent study which examined cheek kissing in humans in 10 cities of France demonstrated that social pressures are involved in modulating laterality at the individual or population level^24^. Though cheek kissing and lip kissing are different in terms of movement, force, emotion, and meaning^24^ the mechanisms through which social pressures might act upon the individuals to determine this laterality are possibly the same. However, this should not be confused with the leftward head-turning bias in parent-child kissing which is also thought to be learned from society^22^, but through a different kind of mechanism – cradling infants or babies using the left arm by parents^25–27^.

Although prior studies have made important contributions to the understanding of laterality in kissing, a limitation is that they did not investigate the roles of kiss initiators and kiss recipients in producing the head-turn during kissing. Studies that investigated kissing in romantic couples present findings of ecological significance^18, 19, 23^, but thus far have failed to disentangle the influence of the kiss initiators on the kiss recipients' head turn. Studies that examined laterality in a laboratory setting have taken a step forward methodologically by introducing dolls or plastic heads as neutrally valenced kiss recipients^19–21^. This approach enabled researchers to examine kissing behavior in a laboratory setting by excluding the influence of one kissing partner upon another, but at the same time, it made them unable to examine head-turning bias in kiss recipients and the true nature of head-turning among the kissers in such an artificial non-emotional situation. Kissing is a form of non-verbal communication, a kind of tactile intimacy, characterized by lively romantic-sexual interactions made by lip-to-lip contact between the partners of a couple. Related to this, prior research has shown significant sex differences in other types of sexual intimacy or heterosexual interactions (e.g., sexual dating, sexual initiation), with males playing the more dominant role of the initiator, more often than females, according to studies conducted in some Western^28, 29^ and rich liberal Asian^30^ societies, that is, W.E.I.R.D. (Western, Educated, Industrialized, Rich, and Democratic) societies^31^. This point reiterates the need for further research including non-W.E.I.R.D. participants in order to understand human nature more broadly. It is likely that such a male bias also happens in the initiation of kissing, and could be even more pronounced in participants of a non-W.E.I.R.D. patriarchal (male-dominated)^32^ conservative Muslim society, like Bangladesh. Because kisses are rarely shared between an adult male and an adult female in public in this society it is very difficult to anticipate the social influences, if any, on the development of laterality during kissing. A benefit of studying this population is that perhaps there will be far less influence of social observation and conformity given the private nature of kissing there. However, it is commonly known that people in this Asiatic society predominantly read and write in a left–to–right direction which, according to Shaki’s^23^ cultural shaping theory, might be associated with laterality in kissing. But, data from such a society are lacking. Thus these aspects of laterality in kissing have been partially and poorly understood, warranting a study in such a non-W.E.I.R.D. patriarchal society^31^.

In addition to cultural representation, another main concern here is the methodology of studying such face-to-face romantic-sexual interactions. We argue that studying the interactions of a kissing pair might only demonstrate the head-turning bias of the dominant partner who initiates the kiss: There will be a first kisser, first turner! The direction of the kiss initiator’s head-turning to a particular side, which we call spontaneous turning in the first single kiss, will exhibit the actual head-turning bias of that partner, but this will not tell us anything about the spontaneous and actual head-turning direction of the kiss recipient. The kiss initiator might indeed have a bias to turn the head to the right, but at that point the kiss recipient (who might not actually be a right-turner) might do the same submissively in order to avoid any potential discomfort which might be felt upon turning the head to the opposite side (i.﻿e., orienting at the same line in space). This indicates that the head-turning direction of the kiss recipient might not always be in a spontaneous direction, the direction he/she would prefer if he/she were to initiate the kiss. The spontaneous head-turning bias can thus be estimated for only one partner in a kissing pair, unless otherwise the two partners are biased to turn their heads to the same side (in reference to self). The two partners possibly cannot turn their heads to the opposite sides simultaneously; turning the two heads to the opposite sides means orienting them at the same line in space which is perhaps conflicting and inconvenient for enjoying the act of kissing. So, when a right-turner kisses a left-turner first, the left-turner will possibly turn the head to the right– even though it is not the preferred direction, and similarly when a left-turner kisses a right-turner first, the right-turner will also likely turn the head to the left. Thus, in a kissing pair comprising partners of spontaneous head-turning biases in the opposite directions (i.e., one partner is a right-turner and another partner is a left-turner) the spontaneous head-turning direction of the kiss initiator is likely to alter the spontaneous/preferred head-turning direction of the kiss recipient. However, such an impact of one partner on the other cannot be anticipated in a kissing pair comprising partners of spontaneous head-turning biases in the same direction (i.e., both the partners are right- or left-turners).

It follows from the above line of reasoning that Güntürkün’s^18^ data indicate that 32.26% (80) of the kissing individuals (248) made a spontaneous head-turn to the right and 17.74% (44) made a spontaneous head-turn to the left, while the remaining 50% (124) of the kissing individuals’ spontaneous head-turning direction was not actually examined. Similarly, the spontaneous head-turning direction of 50% of the kissing individuals (the kiss recipients) was not studied in any other prior studies on kissing couples^19, 23^ as they used a method which was oriented solely on the behavior of the kiss initiators. Thus 100% of the kiss recipients’ (spontaneous) head-turning direction was, in fact, unknown in all the prior studies, indicating that the findings they reported were erroneous being that an important aspect – the recipient behavior – was missing. Besides the previously unexamined behavior of the kiss recipients, a number of other variables including sex and handedness that can potentially modulate the initiation of kissing and head-turning bias in kissing have not been studied systematically. Males and females are different in many ways, and such differences can vary as a function of handedness^33–36^, prenatal hormone levels^37^ and dopaminergic levels in the brain^38–40^. A mounting body of evidence suggests that these factors can potentially modulate sexual dominance, romantic interactions, and behavioral laterality in humans^17, 41, 42^. For example, testosterone, an endogenous hormone, which is higher in males than females, regulates sex drive (libido in humans, courtship behavior in animals)^42^ and appears to germinate seeds of personality^43^; a higher level of testosterone makes the person more dominant, sexually more active and aggressive^41^. In face-to-face emotional or social interactions, testosterone can also play the role of promoting status-seeking behavior by a modulation of reward processing and motivational drive in the dopaminergic system^41, 44, 45^. Beyond the influence of testosterone, dopamine (DA, a neurotransmitter) has also its unique role to modulate leaning or turning behavior perhaps via handedness in both model organisms and humans^17^.

Here we formulate three hypotheses grounded on the above research findings. First, we hypothesize that there is a sex difference in kissing – typically males play the role of an initiator and females play the role of a passive recipient. A second hypothesis is that handedness determines head-turning direction, particularly spontaneous head-turning direction, in the first kiss which can typically be observed and tested in the partners who initiate the kiss rather than those who receive it. Related to this is our third hypothesis which states that irrespective of sex and handedness the head-turning direction of the kiss recipients is modulated by the head-turning direction of the kiss initiators. In order to test these novel hypotheses, overcome the limitations of prior studies (see above), and extend the research to a new understudied non-W.E.I.R.D. population^31^, we assessed 51 heterosexual married couples in Bangladesh. Indeed, this is the first study on laterality in kissing in a private naturalistic context that eliminates the effects of an observer or public on such an intimate form of physical interactions. Data about this kind of private romantic behavior from such a non-W.E.I.R.D. patriarchal conservative Muslim society which is often overlooked in scientific enquiry^31^ advance our understanding of the aspects of kissing and lateral bias in kissing behavior in adult humans.

## Results

**Figure 1:**
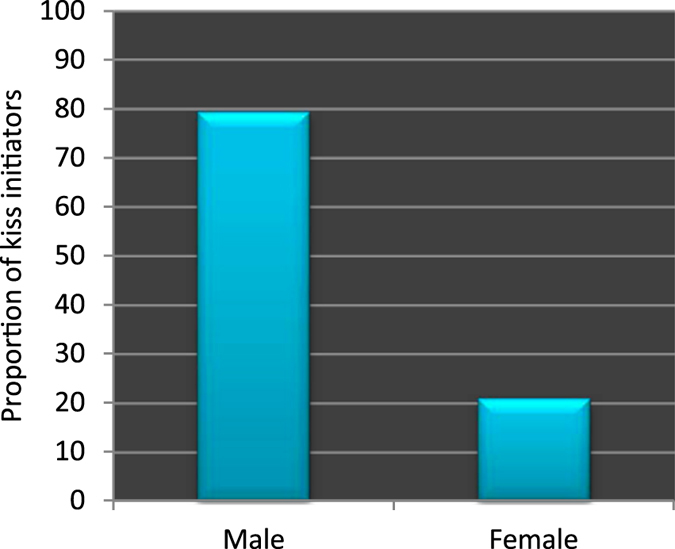
Proportions of males and females initiating a kiss.

First, we calculated the proportion of males and females initiating a kiss. We observed that 79.17% of the kiss initiators were males (Figure 1), and that the ratio of males and females initiating a kiss was significantly different from 50% (*χ* ^*2*^(1) = 16.33, *p* < 0.001). This has further been corroborated by the outcomes of a binary logistic regression where we examined the effects of sex and handedness on the likelihood that participants have a bias in the initiation of kissing. The logistic regression model was significant (*χ* ^*2*^(2) = 34.878, *p* < 0.001). The non-significant value of the Hosmer and Lemeshow test (*p* = 0.993) suggests that our model is a good fit to the data. The model explains 40.60% (Nagelkerke *R* ^*2*^) of the variance in the initiation of kissing. Table 1 displays the logistic regression coefficient (*B*), *Wald*, and *Odds Ratio* (*Exp*(*B*)) for each of the two explanatory variables. It shows that there is a significant effect of sex (*Wald* = 28.186, *df* = 1, *p* < 0.001), but not the effect of handedness, on the initiation of kissing. The *Odds Ratio* for sex indicates that when holding handedness constant males are 14.532 times more likely to initiate kissing as opposed to females.

**Table 1:** Effects of sex and handedness on the initiation of kissing demonstrated through a binary logistic regression.

Variable	*B*	*SE*	*Wald*	*df*	*p*	*Exp(B)*	95% *CI* for *Exp(B)*
(Intercept)	−1.246	0.536	5.397	1	0.020	0.288	
Sex	2.676	0.504	28.186^*^	1	< 0.001	14.532	5.410–39.031
Handedness	−0.001	0.005	0.048	1	0.827	0.999	0.988–1.009

Note. *N* = 96.

Coding independent variable, Sex: Male = 1.000; Female = 0.000 (Reference category).

Coding dependent variable, Initiation of kissing: No = 0; Yes = 1.

**Figure 2:**
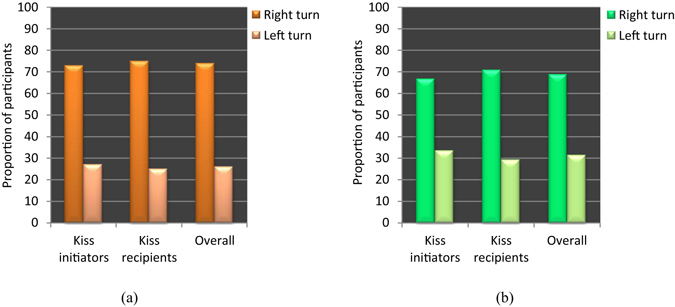
Proportions of participants turning head to the right or left during kissing. (**a**) Data provided by kiss initiators. (**b**) Data provided by kiss recipients. Two sets of head-turning data were obtained because each participant reported about the direction of his or her own head-turning as well as the direction of his or her partner’s head-turning while initiating or receiving a kiss, but without having any knowledge of the partner’s response.

Second, we calculated the proportion of participants turning the head to the right and that of those turning the head to the left during kissing. Kiss initiator provided data revealed that 72.92% of the kiss initiators and 75% of the kiss recipients turned their heads to the right (Figure 2a), the ratio of the right- and left-turners in each category being significantly different from 50% (*χ* ^*2*^(1) = 10.083, *p* = 0.001 for kiss initiator category; *χ* ^*2*^(1) = 12.000, *p* = 0.001 for kiss recipient category). Consistently, kiss recipient provided data revealed that 66.67% of the kiss initiators and 70.83% of the kiss recipients turned their heads to the right (Figure 2b), and that the ratio of the right- and left-turners in each category was significantly different from 50% (*χ* ^*2*^(1) = 5.333, *p* = 0.021 for kiss initiator category; *χ* ^*2*^(1) = 8.333, *p* = 0.004 for kiss recipient category). Similar results were demonstrated when the data were analyzed again by taking all of the participants together irrespective of who initiated or received the kiss. Overall, 68.75% (kiss recipient provided) to 73.96% (kiss initiator provided) of the participants turned their heads to the right (Figure 2), and the ratio of the right- and left-turners was significantly different from 50% (*χ* ^*2*^(1) = 13.500, *p* = 0.001 for kiss recipient provided data; *χ* ^*2*^(1) = 22.042, *p* = 0.001 for kiss initiator provided data).

In order to see the effects of sex and handedness on such a bias during kissing, we performed binary logistic regressions on the collective head-turning data for the kiss initiators and the kiss recipients (Table 2). The overall logistic regression model was significant (*χ* ^*2*^ (2) = 7.876, *p* = 0.019 for kiss initiator provided data) or showed a tendency in the direction of significance (*χ* ^*2*^(2) = 5.422, *p* = 0.066 for kiss recipient provided data). The non-significant values of the Hosmer and Lemeshow test suggest that our models are a good fit to both the kiss initiator provided data (*p* = 0.437) and the kiss recipient provided data (*p* = 0.771). The overall model for the kiss initiator provided data explains 11.50% (Nagelkerke *R* ^*2*^) of the variance in head-turning bias, and the overall model for kiss recipient provided data explains 7.70% (Nagelkerke *R* ^*2*^) of the variance in head-turning bias. Table 2 shows that there is a significant effect of handedness on head-turning bias as demonstrated by the outputs for both the kiss initiator provided data (*Wald* = 6.856, *df* = 1, *p* = 0.009) and the kiss recipient provided data (*Wald* = 4.732, *df* = 1, *p* = 0.030). The *Odds Ratio* for handedness indicates that when holding sex constant, an increase in handedness score by 1 unit (increased right-handedness) increases the likelihood of overall head-turning bias to the right by 1.013 times (for kiss initiator provided data) or 1.010 times (for kiss recipient provided data). However, the impact of participants’ sex on head-turning bias was not significant.

**Table 2:** Effects of sex and handedness on the overall head-turning direction demonstrated through binary logistic regressions.

Variable	*B*	*SE*	*Wald*	*df*	*p*	*Exp*(*B*)	95% *CI* for *Exp*(*B*)
Data provided by kiss initiators							
(Intercept)	0.169	0.496	0.116	1	0.734	1.184	
Sex	−0.178	0.490	0.132	1	0.717	0.837	0.320–2.188
Handedness	0.013	0.005	6.856^*^	1	0.009	1.013	1.003–1.023
Data provided by kiss recipients							
(Intercept)	0.124	0.479	0.067	1	0.795	1.132	
Sex	−2.51	0.456	0.302	1	0.583	0.778	0.318–1.903
Handedness	0.010	0.005	4.732^*^	1	0.030	1.010	1.001–1.020

Note. *N* = 96.

Coding independent variable, Sex: Male = 1.000; Female = 0.000 (Reference category).

Coding dependent variable, Head-turning direction: Left = 0; Right = 1.

Third, we also performed binary logistic regressions to see the impacts of sex and handedness on the kiss initiators’ head-turning bias and the kiss recipients’ head-turning bias separately. The regression model for the kiss initiators’ head-turning data was significant (*χ* ^*2*^(2) = 7.100, *p* = 0.029 for kiss initiator provided data) or showed a tendency in the direction of significance (*χ* ^*2*^(2) = 4.698, *p* = 0.095 for kiss recipient provided data). As revealed by Nagelkerke *R* ^*2*^ values, the models explained 12.90% (kiss recipient provided data) to 20.00% (kiss initiator provided data) of the variance in head-turning bias in the kiss initiators. As depicted in Table 3, results further showed that the effect of handedness on head-turning bias was significant (*Wald* = 4.591, *df* = 1, *p* = 0.032 for kiss initiator provided data) or showed a tendency in the direction of significance (*Wald* = 3.459, *df* = 1, *p* = 0.063 for kiss recipient provided data) in the kiss initiators. The *Odds Ratio* for handedness indicates that when holding sex constant, an increase in handedness score by 1 unit (increased right-handedness) increases the likelihood of turning head to the right by 1.019 times (for kiss initiator provided data) or 1.015 times (for kiss recipient provided data). However, the regression models for the kiss recipients’ head-turning data provided by both kiss initiators and kiss recipients fell short of statistical significance (data not shown).

**Table 3:** Effects of sex and handedness on the kiss initiators’ head-turning direction demonstrated through binary logistic regressions.

Variable	*B*	*SE*	*Wald*	*df*	*p*	*Exp(B)*	95% *CI* for *Exp(B)*
Data provided by kiss initiators							
(Intercept)	−0.396	1.011	0.154	1	0.695	0.673	
Sex	−0.109	0.891	0.015	1	0.903	0.897	0.157–5.137
Handedness	0.019	0.009	4.591^*^	1	0.032	1.019	1.002–1.038
Data provided by kiss recipients							
(Intercept)	−0.094	0.922	0.011	1	0.918	0.910	
Sex	−0.463	0.849	0.297	1	0.585	0.629	0.119–3.322
Handedness	0.015	0.008	3.459	1	0.063	1.015	0.999–1.031

Note. *N* = 48.

Coding independent variable, Sex: Male = 1.000; Female = 0.000 (Reference category).

Coding dependent variable, Kiss initiator’s head-turning direction: Left = 0; Right = 1.

Then we performed another binary logistic regression, adding the kiss initiators’ head-turning direction as a third explanatory variable of the kiss recipients’ head-turning direction (Table 4). This analysis was done for the kiss initiator provided data only because the other set of data (kiss recipient provided) included some outliers. Results showed that the new model for the kiss initiator provided data was significant (*χ* ^*2*^(2) = 12.230, *p* = 0.007), indicating that the model with the third explanatory variable is a significant improvement not only over the null model, but also over the model which was non-significant with sex and handedness as the explanatory variables. The non-significant value of the Hosmer and Lemeshow test (*p* = 0.554) suggests that the new model is a good fit to the data. The model explains 33.30% (Nagelkerke *R* ^*2*^) of the variance in kiss recipients’ head-turning bias. Table 4 shows that there was a significant effect of the kiss initiators’ head-turning direction on the kiss recipients’ head-turning direction (*Wald* = 9.382, *df* = 1, *p* = 0.002), but the effects of the kiss recipients’ sex and handedness were non-significant. The *Odds Ratio* for the kiss initiators’ head-turning direction indicates that when holding the kiss recipients’ sex and handedness constant, a rightward head turn by the kiss initiators is 11.327 times more likely to alter the kiss recipients’ head turn to the right, as opposed to a leftward head turn by the kiss initiators altering the kiss recipients’ head turn to the left.

**Table 4:** Effects of the kiss recipients’ sex, handedness, and the kiss initiators’ head-turning direction on the kiss recipients’ head-turning direction demonstrated through a binary logistic regression on the kiss initiator provided data.

Variable	*B*	*SE*	*Wald*	*df*	*p*	*Exp(B)*	95% *CI* for *Exp(B)*
(Intercept)	−0.695	0.805	0.745	1	0.388	0.499	
Kiss recipients’ sex	−0.264	0.956	0.076	1	0.782	0.768	0.118–4.997
Kiss recipients’ handedness	0.004	0.008	0.333	1	0.564	1.004	0.989–1.020
Kiss initiators’ head-turning direction	2.427	0.792	9.382^*^	1	0.002	11.327	2.397–53.533

Note. *N* = 48.

Coding independent variable, Sex: Male = 1.000; Female = 0.000 (Reference category).

Coding independent variable, Kiss initiator’s head-turning direction: Right = 1.000; Left = 0.000 (Reference category).

Coding dependent variable, Kiss recipient’s head-turning direction: Left = 0; Right = 1.

**Figure 3:**
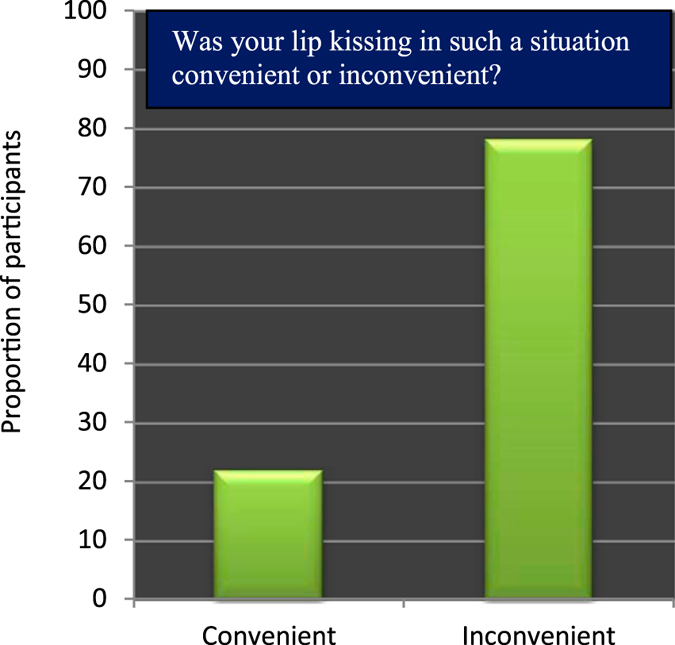
Proportions of participants who perceived kissing, with the two heads turned to the opposite direction (i.e., oriented at the same line in space), as convenient or inconvenient.

The dependence of the kiss recipients’ head-turning direction on the kiss initiators’ head-turning direction was further corroborated by the evidence that 78.12% of the participants reported kissing with the two heads turned to the opposite direction (i.e., oriented at the same line in space) as inconvenient (Figure 3). The ratio of this (inconvenient) proportion of participants to the remaining who reported such a kissing situation as convenient was significantly different from 50% (*χ* ^*2*^(1) = 30.375, *p* < 0.001). By further analyzing the data in a binary logistic regression we did not find any evidence that this difference can be accounted for sex or handedness of the participants. Based on these findings we conclude that despite the normal tendency of handedness to modulate head-turning direction during kissing the kiss initiators’ head-turning direction can modify or alter that effect to a comfortable direction in the kiss recipients.

## Discussion

This study demonstrated some novel and interesting aspects of kissing behaviors and laterality in adult humans. Specifically, we found that there was a significant male bias (14.532 times more likely than females) in the initiation of kissing (Table 1, Figure 1), and a significant rightward bias in head-turning in both the kiss initiators and the kiss recipients. The rightward head-turning bias in the kiss initiators was found to be associated with their handedness (Table 3), and that in the kiss recipients was found to be associated with the kiss initiators’ head-turning direction (Table 4). Though there is no prior study to directly compare the male bias in the initiation of kissing it is consistent with other relevant studies which showed that males are dominant over females in other forms of sexual activities, such as sexual dating, and sexual initiation^28–30^. The rightward bias in head-turning during kissing is consistent with a similar bias in whole body turning in humans^17, 46–48^. The proportions of the right- and left-turners in the act of kissing (Figure 2) also closely correspond to the proportions reported in prior studies^18, 19, 23^. The impact of handedness on the spontaneous head-turning direction in the kiss initiators supports the postulate that the head-turning bias in kissing is related to handedness^20^ though there is also an opposite view^18, 23^. The impact of the kiss initiators’ head-turning direction on the kiss recipients' head-turning direction during kissing is in line with the evidence that participants felt discomfort while engaged in the act of kissing with the two heads turned to the opposite directions (i.e., oriented at the same line in space; Figure 3). This latter finding leads us to argue that irrespective of sex and handedness, the kiss recipients showed a tendency to match their partners’ head-turning direction during kissing to avoid discomfort.

An inspection of our results indicates that the regression models with sex and handedness explain more of the variance in head-turning bias (Nagelkerke *R* ^*2*^: 12.90–20.00%) in the kiss initiators than the variance they explain (Nagelkerke *R* ^*2*^: 7.70–11.50%) in overall head-turning bias (i.e., when data for the kiss initiators and the kiss recipients were analyzed collectively). The *Odds Ratio* showed that the predictability of handedness increased when the kiss initiators’ head-turning bias was examined separately from the kiss recipients’ head-turning bias (*Exp*(*B*): 1.015–1.019; Table 3) than when they were analyzed collectively (*Exp*(*B*): 1.010–1.013; Table 2); yet either way the ﻿*Odds Ratios*﻿ suggested that there was not a strong effect overall in these cases. Consistent with the low *Odds Ratios*, the regression models with the same two explanatory variables were non-significant for the kiss recipients’ head-turning data. But, when the kiss initiators’ head-turning direction was taken as a third explanatory variable in the regression model we found that the new model is significant, and explains a large amount of the variance (Nagelkerke *R* ^2^: 33.30%) in their head-turning bias. Here the *Odds Ratio* showed that a rightward head turn by the kiss initiators is 11.327 times more likely to alter the kiss recipients’ head turn to the right, as opposed to a leftward head turn by the kiss initiators altering the kiss recipients’ head turn to the left. These findings lead us to reiterate the argument that the kiss recipients’ head-turning direction is modulated by the kiss initiators’ spontaneous head-turning direction (see Introduction), and therefore it is not appropriate to analyze the head-turning data for both partners in a collective fashion as done by prior studies^18^. Thus we suggest that in order to better understand the lateral bias in kissing one should separately examine the data for the kiss initiators and those for the kiss recipients.

At the outset, societal learning or cultural norms can account for the findings reported here. In most traditional societies, males are expected to take on the more dominant role in sexual interactions while females are typically expected to be responsive to males’ desires and wait for them to initiate and orchestrate sexual activities^28, 29, 49, 50^. This impact of socialization is perhaps more intense in a conservative Muslim society like Bangladesh where religious norms restrict females to be less sexually active and more submissive as opposed to males^51–53^. These male-favoring religious or cultural norms are commonly and often strictly followed in both indoor and outdoor activities^32^. It has been suggested that females have a greater level of erotic plasticity (changing nature of sex drive) than males due to these sociocultural influences^51–53^. Perhaps this has been reflected in the act of kissing by the participants in our study. Secondly, people in this Asiatic society typically have a habit of scanning information (e.g., reading and writing text, drawing pictures) from a left to right direction which might cause them to turn their heads to the right while engaged in kissing with romantic partners. As in other Asiatic and Muslim societies, most of the people in this society have a right-handed tendency in daily activities (the estimated proportion is about 92% in this study; see Methods section), and therefore we contend that the association of right-handedness with the rightward head-turning bias during kissing might have been further strengthened by the traditional cultural spatial habits.

However, cultural norms or spatial habits might not be the only factors to exclusively modulate the male bias in the initiation of kissing or the rightward bias in head-turning during kissing. An in-depth review of the literature reveals that the male-favoring cultural or societal norms of sexual initiation in Bangladesh are in line with the differences in biological makeup between males and females, and the right-hand bias in head-turning during kissing is in line with the potential biological differences between the right- and left-handers. Specifically, one potential biological factor that might interact with the cultural or societal norms to modulate the male bias in the initiation of kissing is endogenous testosterone. Research has shown that testosterone is involved in regulating sex drive^41, 42^, and also appears to affect face-to-face emotional interactions and social status-seeking motives or behavioral dominance^41, 44, 45^. How could this hormone affect face-to-face emotional interactions during kissing? Few data are available to answer this question. It has been suggested that baseline testosterone reflects a person’s personality traits^43^, a higher level of testosterone indicating that the person is sexually more dominant and aggressive^41^. Relevant to this, neuroimaging studies have shown that testosterone enhances amygdala activation in both males^54^ and females^55, 56^. In a face-to-face situation, when the two partners are spatially close to each other they work as sexual social stimuli for each other which induce their testosterone to a larger scale relative to the baseline^57, 58^. Because of a preexisting innate discrepancy in the amount of testosterone between males and females it is plausible that the elevation of this hormone level in such a romantic situation is much higher in the male than the female partner and that this can differentially affect their amygdala activation (though it has not yet been tested). Thus at the preparatory stage of the act of kissing, the increased level of testosterone perhaps motivates the male partner to take it as a challenge^59^, and to assert power. The female partner, due to a lower testosterone level is instead the passive recipient by this account.

Reward processing is a crucial element in emotional or social interactions^60^ and social hierarchies^61^ and can be influenced by testosterone. It has been suggested that testosterone might promote status-seeking behavior by a modulation of reward processing and motivational drive in the DA system^41^. Thus a second potential candidate in the biological realm that can explain the male bias to initiate kissing is DA which has a variety of dominant behavioral or motivational functions^62^ and can vary across sexes. Research has shown that DA modulates pleasure seeking behaviors, such as sex, in active males^63–67^ rather than receptive females^68–70^. In line with this, studies in humans^38–40, 71^ have shown that DA levels are markedly greater in males than females. Taken together, we conclude that males were likely more active, and more motivated, to initiate kissing than females perhaps due to the imbalance of testosterone and DA levels between males and females.

In addition to the modulation of motivational or pleasure seeking behaviors, DA also plays a key role in the development of spatial attentional or orienting bias and directional bias in turning or rotational behaviors^17, 72–75^. Research in both healthy humans and patients with neurological disorders (e.g., Parkinson’s disease) has suggested that presumably, left-handers have greater dopaminergic content in the right hemisphere, whereas for right-handers it is greater in the left hemisphere^13, 76, 77, 79, 80^; however, this inter-hemispheric imbalance of DA cannot be associated with sex. Thus we contend that during kissing in a normal condition participants having greater right-handedness might have shown a greater tendency to turn their heads to the right, and those with greater left-handedness might have shown a greater tendency to turn their heads to the left in our study. However, this biological factor-based interpretation of our results does not necessarily undermine the role culture or society plays in shaping the directional bias in head or whole body turning in humans. To reiterate, a recent study has suggested that head-turning bias during kissing is an acquired behavioral asymmetry, probably shaped by spatial experience, such as reading or writing habits^23^. On the other hand, Scharine and McBeath’s^81^ study suggested that the directional bias in walking is an additive function of both learning (e.g., driving practice) and genetic handedness. Thus though inter-hemispheric imbalance of DA is an innate brain property, it is not resistant to behavioral modification; rather it can change or even alter reversely due to explicit or implicit societal learning or cultural habits.

According to the above discussion, the lateral bias in kissing (in the form of head-turning) develops in a dynamic fashion where the neurogenetic factors (e.g., DA, handedness) and cultural factors (e.g., reading or writing direction) may interplay to determine the direction and extent of bias – a view that finely fits with our recent dynamic model that accounts for the development of a rightward (clockwise) versus a leftward (anticlockwise) bias in visuospatial functioning in general and turning behavior in particular^17^. However, the impact of the kiss initiators’ head-turning direction on the kiss recipients’ head-turning direction demonstrated in this study leads us to argue that the head-turning bias developed in such a dynamic manner may not always be apparent in behavioral expression or may be apparent in a reversed direction depending on the immediate environmental situation^17^. Thus in our study the kiss recipients’ tendency to match their partners’ head-turning direction during kissing might have been to avoid discomfort t﻿hat could potentially be felt upon tur﻿n﻿ing the head to the opposite side (i.e., ﻿orienting at th﻿e sa﻿me line in space).

In conclusion, following an ecologically valid approach we examined lateral bias and other aspects of lip kissing behavior in heterosexual married couples in Bangladesh. As noted earlier in this paper, Bangladesh is a non-W.E.I.R.D.^31^ and patriarchal^32^ conservative Muslim society, where kissing is considered very private and is not typically allowed in public places^82^. It was therefore preferred for us to not directly observe this, and at this stage we could not yet make physiological recordings that directly assess the testosterone and DA hypotheses of kissing and lateral bias when the partners of each couple were freely engaged in kissing each other a﻿t home,﻿ a very private naturalistic setting. Secondly, until this study, there were no direct studies on the neural correlates of kissing or lateral bias in kissing shared by the members of a romantic couple. So, backed by the relevant prior studies and our recent theoretical work^17^, we propose here a number of explanatory conjectures (see above) about the potential associations of testosterone and DA with the initiation of kissing and lateral bias in kissing, within the context of socio-cultural milieu. These are speculative, but reasonable in terms of their known functions and distributions across sexes and across hemispheres (see above). Thus the exact causative factors of the male bias in the initiation of kissing and rightward bias in head-turning during kissing are still unclear and merit further investigation. So, if feasible, future studies can attempt to measure the two suggested neurogenetic factors (testosterone and DA) in the kissing couples, and directly associate them with the initiation of kissing and head-turning direction during kissing. This will enable scientists to delineate the roles testosterone and DA play in the initiation of kissing and lateral bias in this very private behavior. However, another shortcoming of this study is that only 7.29% of the participants sampled had a left-handed tendency (an LI score < 0, see Methods section). Perhaps this is why handedness was found to explain (though significantly) just a small part of the variance in head-turning bias (up to 20.00%; see Results section). So, future replication studies on a sample including more participants of a left-handed tendency may provide sufficient data to test the handedness hypothesis about the lateral bias in kissing behavior (i.e., can explain more variance in head-turning bias). A related and final limitation of this study is the reliance on a sample of small size. Though the sample size was justified based on a review of the relevant prior studies and was good enough for logistic regression analysis (see Methods section), future replication studies with a large scale sample can confirm the present findings.

## Methods

All methods were carried out in accordance with the Declaration of Helsinki guidelines^83^, and in accordance with the legal requirements of Bangladesh and the institutional protocols for research at the University of Dhaka. All participants provided informed verbal consent before participation.

### Participants and measures

We selected 51 heterosexual married couples purposively from the city of Dhaka. They were highly educated (Bachelor or Master’s degree) and opportunistically selected in a convenient manner from a series of key locations, such as an office lounge, a university lounge, and socio-cultural gatherings (wedding anniversaries, birthday parties) of our friends and relatives. The sample size (*N* = 102; 51 males and 51 females) was justified based on a review of the relevant literature. Prior studies on kissing and head-turning behavior used various sample sizes, ranging from 57 to 248 individuals^18, 21^. To administer the survey on our participant group we used two measures, a Head Turning Questionnaire (HTQ, designed in this study) and a Bangla version (translated and modified) of the Edinburgh Handedness Inventory (EHI)^84^. The HTQ comprises 6 items with a binary response (Me/My Partner or Left/Right or Convenient/Inconvenient). One of the items measures spontaneous initiation of kissing (In that romantic situation, who was the first kisser?), two items measure head-turning direction in both the partners during spontaneous kissing (responded by the kiss initiator or first kisser; If you were the first kisser, (a) which direction did you turn your head to on the first kiss? (b) which direction did your partner turn his/her head to while receiving your first kiss?), two items measure head-turning direction in both the partners during spontaneous kissing (responded by the kiss recipient; If your partner was the first kisser, (a) which direction did he/she turn his/her head to on the first kiss? (b) which direction did you turn your head to while receiving his/her first kiss?), and the last one item measures the perceived quality of the act of kissing when the two heads were oriented at the same line in space (Was your lip kissing in such a situation convenient or inconvenient?).

### Procedure

The questionnaires were distributed to the selected couples in closed envelopes. Each couple was provided with two envelopes, one for a husband and one for a wife. Each envelope contained two questionnaires— the HTQ and the BEHI (Bangla EHI). Each of the two questionnaires included standard instructions for the participants to read silently and privately before answering the items. Following the instructions, the members of each couple independently answered the first five items of the HTQ immediately once after finishing the course of spontaneous lip kissing as they do naturally in a face-to-face and standing situation at home, and to answer the last item after sharing a lip kiss with each other, following another course of lip kissing, in a guided manner. That is, before answering the last item, the members of each couple shared a lip kiss with each other for a while in a face-to-face and standing situation, turning one partner’s head in a direction opposite to the turning direction of another partner’s head, the direction of head-turning being defined in reference to each partner’s self. Thus if one partner turned the head to her/his right the other partner turned the head to his/her left and vice versa, such that both their heads were oriented at the same line in space during lip kissing. In any kissing situation (spontaneous or guided), the members of each couple were not allowed to hold any objects in their hands during lip kissing and to discuss or compare any answers with each other prior to completing the questionnaire. Thus each individual reported about the direction of his/her own head-turning as well as the direction of his/her partner’s head-turning while initiating or receiving a lip kiss, but without having any knowledge of the actual responses made by the other partner prior to finishing. In this way we got two sets of head-turning data for all the participants (kiss initiator provided data and kiss recipient provided data). After finishing the HTQ the members of each couple independently filled out the BEHI following the standard instructions provided on it, and returned both the questionnaires to the survey administrator in a closed envelope. Thus data collection from all the participants was finished approximately in three months. Then to process the data for statistical analysis we checked the participants’ responses to both the questionnaires, in particular, we cross-checked the participants’ responses as they reported in the HTQ who initiated kissing. Three couples were excluded from further processing and statistical analyses due to providing inconsistent and/or incomplete responses.

### Data analyses

The BEHI comprises 15 items. The responses of each participant to this inventory were used to estimate his/her Laterality Index (LI) using the formula: LI = (RH − LH)/T × 100 (RH = Number of tasks done with the right hand, LH = Number of tasks done with the left hand, T = Total number of tasks/items). The value of LI on the full inventory ranges from −100 (extreme left-handedness) to +100 (extreme right-handedness). The actual LI scores of our participants also ranged from −100 to +100, with only 7.29% of the participants having an LI score <0, and 91.67% of them having an LI score >0, indicating that most of the participants had a right-handed tendency. However, unlike prior studies^19–21, 23^ that divided handedness data into a couple of groups (left-handed, right-handed) we retained participants’ handedness data in their original, continuous format. In order to code the data measured by the HTQ we reformatted the original binary response ‘Me/My Partner’ as ‘Yes/No’ and also Convenient/Inconvenient as ‘Yes/No’. We coded participants’ sex, initiation of kissing, self-reported head-turning direction, and (perceived) convenience/inconvenience (of lip kissing with the two heads oriented at the same line in space) as dummy variables. Then we studied interdependence of all these variables by computing a correlation matrix (data not shown). The purpose of this initial analysis was to have an overall idea of the nature of our data.

The main analyses were done using a χ^2^-test and binary logistic regression. Because our criterion or dependent variables (initiation of kissing, head-turning, convenience) were binary and the explanatory variables were categorical (sex) or continuous (handedness) we chose binary logistic regression analyses to examine the impacts of the explanatory variables on the criterion variables. Though large samples are preferable for a logistic regression analysis, simulation studies have offered a rule of thumb that for stable regression models one requires 10 to 15 observations per explanatory variable^85–88^. A more recent simulation study has suggested that this rule of thumb is too conservative, and that results from any logistic model with the number of observations per explanatory variable ranging from 5 to 9 can be reliable^89^. Some authors have suggested that results from less than 10 observations per explanatory variable should be cautiously interpreted^87, 90^. Briefly, a small sample can be problematic when there are a large number of explanatory variables in the study. However, in our study, there were only 2 or 3 explanatory variables and the number of observations/participants per explanatory variable ranged from 16 to 48. Thus the sample size required for a logistic regression analysis was satisfied in our study. We carried out a series of binary logistic regression analyses (in addition to a *χ* ^*2*^-test where appropriate) after ensuring that the data satisfied the underlying assumptions (absence of collinearity of the explanatory variables, no outliers in the data). Because all the interaction terms fell short of statistical significance we excluded them from the final regression models in order to make the models simple and more comprehensive. Through this analysis our study goes beyond all prior studies that were mostly based on division of data into a few handedness categories, calculating some descriptive statistics (percentages, correlations) and subjecting data to a *χ* ^*2*^-test only^18, 20, 21, 23^.

### Availability of Materials and Data

A copy of the full HTQ designed and used in the current study can be obtained from the corresponding author via email. The datasets generated and analyzed during the study are also available in SPSS and/or Excel format from the same author upon reasonable request.
